# Noninvasive Therapeutic Monitoring of Circulating Tumor DNA in BRAF-Mutant Metastatic Colon Cancer Using Droplet Digital PCR, Next-Generation Sequencing, and Fragmentomics

**DOI:** 10.1155/crom/8140524

**Published:** 2025-11-07

**Authors:** Rachel C. T. Lam, Connie W. C. Hui, Irene O. L. Tse, Qing Zhou, Chit Chow, Wei Kang, K. C. Allen Chan, Brigette B. Y. Ma, W. K. Jacky Lam

**Affiliations:** ^1^Faculty of Medicine, The Chinese University of Hong Kong, Ma Liu Shui, Hong Kong; ^2^State Key Laboratory of Translational Oncology, Sir YK Pao Centre for Cancer, Shatin, Hong Kong; ^3^Cancer Drug Testing Unit, Hong Kong Cancer Institute, Shatin, Hong Kong; ^4^Department of Chemical Pathology, The Chinese University of Hong Kong, Ma Liu Shui, Hong Kong; ^5^Centre for Novostics, Hong Kong Science Park, Ma Liu Shui, Hong Kong; ^6^Li Ka Shing Institute of Health Sciences, The Chinese University of Hong Kong, Ma Liu Shui, Hong Kong; ^7^Department of Anatomical and Cellular Pathology, The Chinese University of Hong Kong, Ma Liu Shui, Hong Kong; ^8^State Key Laboratory of Digestive Disease, The Chinese University of Hong Kong, Ma Liu Shui, Hong Kong; ^9^Department of Clinical Oncology, The Chinese University of Hong Kong, Ma Liu Shui, Hong Kong

## Abstract

**Purpose:**

*BRAFV600E*-mutated metastatic colorectal cancers (mCRCs) are associated with poorer prognosis. We present a case, in which noninvasive therapeutic monitoring was performed on a patient with *BRAF*-mutant mCRC, aiming to track disease progression and elucidate the mechanisms of response and resistance towards anti-*BRAF* therapy.

**Methods:**

A 40-year-old man diagnosed with metastatic *BRAFV600E* mutant sigmoid adenocarcinoma received multiple lines of treatment, including first-line chemotherapy + bevacizumab and targeted therapy of cetuximab, encorafenib ± binimetinib. Noninvasive therapeutic monitoring was performed on ctDNA using our in-house designed droplet digital PCR assay and fragmentomics. We also performed serial and paired analyses of tissue, liquid biopsy, and in vitro studies at different multiple timepoints.

**Results:**

ctDNA and fragmentomics biomarkers were concordant with, and even preceded traditional serological and radiological biomarkers in predicting disease progression. Molecular analyses and drug testing also revealed mutations that are either potentially targetable or account for resistance, which guided the subsequent treatment regimen.

**Conclusion:**

This case demonstrates the potential application of ctDNA and fragmentomics biomarkers, molecular analyses, and drug testing in noninvasive therapeutic monitoring of *BRAFV600E* mutant mCRC. These illustrate the potential application of such noninvasive therapeutic monitoring in larger scale cohorts of patients.

## 1. Introduction

*BRAF* mutations are found in around 12% of metastatic colorectal cancer (mCRC) patients, with *V600E* being the main type, causing constitutional activation of the MAPK pathway and abnormal cell proliferation [1, 2]. As a highly heterogeneous disease entity, the *BRAFV600E* mutation confers a poorer prognosis, with a median overall survival (OS) of less than 20 months. The BEACON trial concluded that triplet therapy (cetuximab + encorafenib + binimetinib) is superior to chemotherapy in terms of survival among patients with *BRAF* mutant mCRC [3, 4], which was approved by the Food and Drug Administration and European Medicines Agency as a second-line therapy.

Here, we present the case of a *BRAF* mutant mCRC, on whom we performed noninvasive therapeutic monitoring by various biomarkers, including circulating tumor (ct) DNA and fragmentomics. We also report the molecular analyses performed for characterization of the patient's response and disease progression during anti-*BRAF* therapy.

## 2. Case Presentation

A 40-year-old man presented with sigmoid adenocarcinoma with metastases to the liver, retroperitoneal and regional nodes in September 2020. Targeted next-generation sequencing (NGS) using the Chinese University of Hong Kong Somatic Mutation v3 Test for Solid Cancers was performed on the primary tumor and identified it to be microsatellite stable (MSS), *RAS*-wild type with *BRAF-V600E, RNF43, TP53*, and *CTNNB1* mutations.

### 2.1. Initial Oncological Treatment

He was treated with first-line FOLFOXIRI + bevacizumab (infusional fluorouracil 400 mg/m^2^ over 30 min on Day 1, 600 mg/m^2^ over 20–22 h on Day 2; folinic acid 200 mg/m^2^ with oxaliplatin 85 mg/m^2^ over 2–3 h on both days; oxaliplatin 85 mg/m^2^ on Day 1; irinotecan 165 mg/m^2^ over 90 min on Day 1 and bevacizumab 5 mg/kg over 30–90 min on Day 1), and achieved complete metabolic response based on a fluorodeoxyglucose positron emission tomography-computed tomography (FDG-PET-CT) following eight cycles of treatment. He was continued with maintenance capecitabine (1800 mg BD on Days 1–14) and bevacizumab (7.5 mg/kg over 30–90 min on Day 1) for 11 cycles, then progressed with rising serum carcinoembryonic antigen (CEA) levels. He was then treated with second-line doublet—cetuximab (500 mg/m^2^ over 60–120 min) and encorafenib (300 mg once daily) as described in the BEACON trial [4], and went into complete remission on FDG-PET-CT after seven cycles of treatment.

### 2.2. Plasma Monitoring of Molecular Response

To track the patient's molecular response to anti-*BRAF* therapy, blood samples were collected before and during treatment at regular intervals for measurement of plasma *BRAF* mutant allele percentage (p*BRAF*%). An in-house droplet digital (dd) polymerase chain reaction (PCR) assay was designed to target *BRAFV600E* mutation in plasma using the QX100/200 System (Bio-Rad, United States), with a sensitivity detection limit of 0.1% and analytical specificity of 100% in normal subjects or controls (*N* = 10). All tissue samples were obtained with the patient's written consent and approved by the Institutional Ethics Committee.

**Figure 1:**
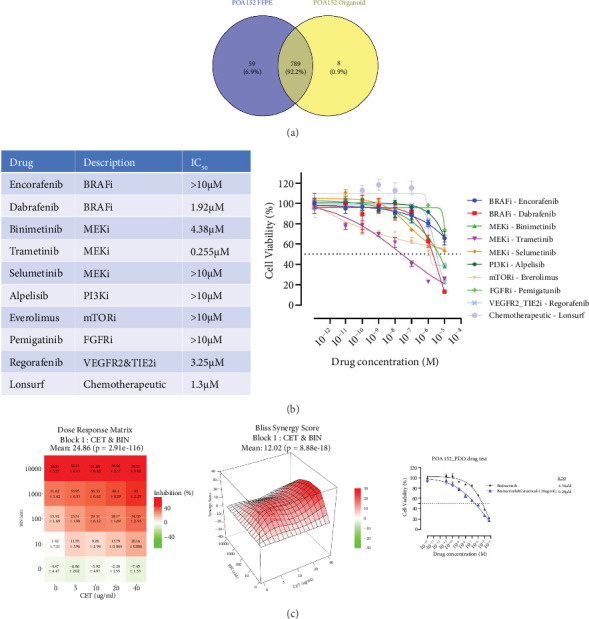
(a) Venn diagram on concordance of common SNPs between primary site and established patient-derived organoids. (b) Cytotoxicity of drugs on PDO treated with drugs for 96 h. (c) Synergy score and dose–response curves showing the inhibitory effect of binimetinib on PDO with and without concomitant cetuximab (20 *μ*g/mL).

Perhaps due to the low tumor burden when the doublet was initiated, p*BRAF*% remained undetectable before and during treatment. He remained on the doublet until FDG-PET-CT 18 months later showed a new solitary liver metastasis, for which he underwent a left lateral liver sectionectomy. Prior to surgery, p*BRAF*% was undetectable, while CEA was 4.5. To elucidate the mechanism of resistance to the doublet, frozen samples of the resected liver metastasis were obtained, and patient-derived organoids (PDOs) were established for drug testing. Paired analysis of germline mutations showed high concordance with the primary tumor, proving that the PDO originated from the primary tumor (Figure 1a). In vitro drug testing was performed on the organoids according to modified STAR protocol [5]. In brief, organoids were mixed with Matrigel and grown for 4 days, then exposed to several drugs in different concentrations for 96 h. CellTiter-Glo 3D (Promega, Medison, United States) was used to assess the drugs' cytotoxicity, and data were analyzed by GraphPad Prism 10 (Figure 1b). The PDOs exhibited a higher IC50 for encorafenib than dabrafenib, implying that the organoids are already resistant to encorafenib. The drug combination effects were assessed by SynergyFinder Plus [6] (Figure 1c) and the cetuximab–binimetinib combination has a high synergy score. A higher potency seen in dose–response curves suggests that the addition of binimetinib could be efficacious clinically.

**Figure 2:**
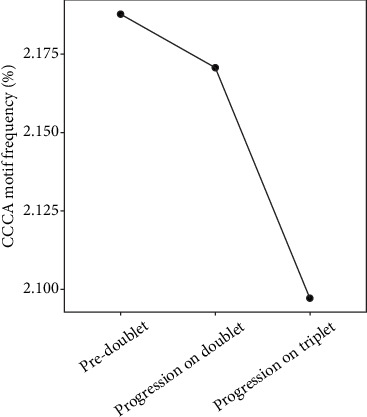
Graph showing CCCA motif frequency among different timepoints.

Other molecular studies were also performed at this timepoint. Whole-exome sequencing (WES) of liver metastasis using Nextseq 500 from Illumina identified *CTNNB1*, *RNF43*, and *RICTOR* mutations (Table 1). NGS on the plasma cell-free DNA using Roche's AVENIO ctDNA Surveillance Kit identified *BRAFV600E* and *PIK3CA* mutations in plasma (Table 2). Fragmentomics analysis was also performed for the measurement of CCCA end motif frequency (Figure 2).

### 2.3. Subsequent Therapy Following Disease Progression With Doublet

p*BRAF*% became detectable via ddPCR at 1 month (0.4%) and 3 months (3.13%) postoperatively (post-op), while CEA remained normal immediately post-op. He was continued on doublet, until FDG-PET-CT and CEA 3 months later revealed rapid disease progression in the primary tumor, liver, and regional nodes. He received bridging therapy of FOLFOXIRI + bevacizumab and then was started on triplet therapy (cetuximab 500 mg/m^2^ over 60–90 min every 3 weeks, encorafenib 300 mg daily, and binimetinib 45 mg BD), which was guided by our PDO drug testing results. He responded clinically with decreasing CEA, and p*BRAF*% became undetectable. Then, 4 months after starting the triplet, p*BRAF*% became detectable again, followed by a rise in CEA. Subsequent FDG-PET-CT confirmed widespread disease progression and p*BRAF*% surged from 4.6% to 52.4% within 2 months. A rebiopsy of his primary tumor was obtained for WES and NGS, which revealed no significant mutations accounting for resistance (Tables 1 and 2). Longitudinal fragmentomics analysis for CCCA end motif frequency was also performed and compared across various timepoints (before starting doublet, progression on doublet, and progression on triplet) (Figure 2).

He was started on dabrafenib (150 mg BD) + trametinib (2 mg daily) without anti-EGFR therapy due to the patient's preference but still developed disease progression in the lungs and liver. Subsequently, he was put on Lonsurf (60 mg BD on Days 1–5, 8–12) ± bevacizumab as a continuation beyond progression, and his disease was static for 3 months, before he rapidly deteriorated and succumbed after 4 years of anticancer therapy.

**Figure 3:**
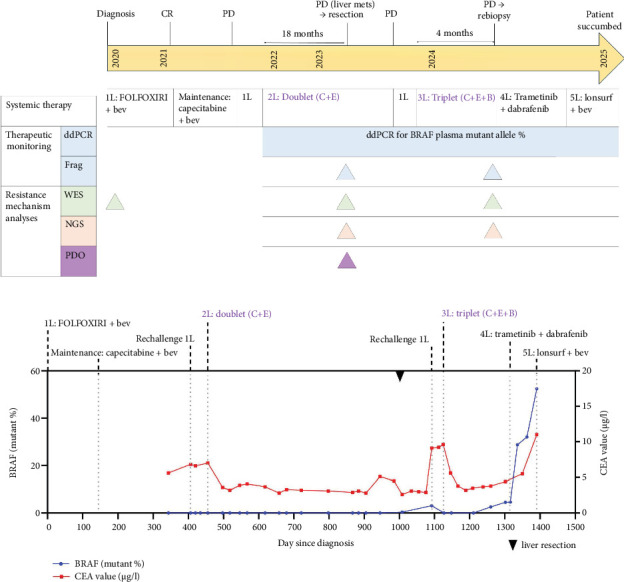
Timeline of treatments received, tests performed ,and comparison of CEA vs. p*BRAF*% across different timepoints. ddPCR, droplet digital polymerase chain reaction; Frag, fragmentomics; WES, whole-exome sequencing; NGS, next-generation sequencing; PDO, patient-derived organoid drug testing; L, line of treatment; Chemo, chemotherapy; Bev, bevacizumab; C, cetuximab; E, encorafenib; B, binimetinib; T, trametinib; D, dabrafenib; CR, complete response; PD, progressive disease.

A summary of our patient's timeline of treatments received, tests performed and comparison of CEA versus pBRAF% across different timepoints could be found in Figure 3.

## 3. Discussion

### 3.1. Biomarkers for Disease Surveillance

ctDNA is a minimally invasive biomarker with potential applications in detecting minimal residual disease (MRD), early recurrence, and guiding treatment options as a predictive biomarker [7]. ddPCR is a well-established method for ctDNA detection with high sensitivity and specificity. It is cost-effective with rapid turnaround time, holding advantages over WES and NGS especially for *BRAF* mutant mCRC with high aggressiveness and rapid disease progression.

We applied our ddPCR assay as a surrogate marker to monitor our patient's tumor burden and predict the efficacy of anti-*BRAF* therapy. Our patient initially had undetectable pBRAF% while on doublet, and he experienced a durable response on doublet (15 months) with a long OS of 51 months. This is in line with the findings of Ros et al., where patients with a high BRAF allele fraction showed worse PFS (hazard ratio [HR] 2.97, 95% confidence interval [CI] 1.55–5.69; *p* = 0.001) and worse OS (HR 3.28, 95% CI 1.58–6.81; *p* = 0.001) than low-BRAF allele fraction patients [8].

Our patient's p*BRAF*% was first detectable post-op for 3 months. Detection of ctDNA post-op is suggestive of MRD, associated with a significantly higher risk of recurrence post-op with a poorer prognosis [9, 10]. It could also be induced by surgical trauma for up to 4 weeks [11]. As ctDNA detection post-op coincided with his progression on doublet radiologically, it is more likely to suggest MRD, predicting nonresponse and disease progression.

Another timepoint of detectable p*BRAF*% was 4 months later while on triplet, followed by a 10-fold increase in p*BRAF*% which corresponded with rapid disease progression clinically, serologically, and radiologically. The rise in p*BRAF*% corresponds to, and even precedes disease progression. It also predicts the increase in tumor burden and subsequent lack of response to anti-*BRAF* therapy, which could allow timely formulation of further lines of treatment guided by molecular studies.

Apart from ddPCR, we also explored the application of fragmentomics biomarkers in plasma DNA for disease surveillance. Cancer-associated fragmentomics profiles (such as size and end motif) have been reported before [12, 13]. Among cancer patients (including CRC), a reduction in the plasma DNA end motif CCCA, the most prevalent 4-mer end motif, was observed. This was postulated to be related to a downregulation of expression of DNASE1L3 in cancers, which is a key nuclease for plasma DNA fragmentation. [13, 14]. Fragmentomics analysis of our patient's plasma by WGS found a decreasing trend in CCCA from predoublet treatment to progression on doublet and triplet, respectively. This reflects an increase in tumor load of our patient throughout these timepoints, which was concordant with his disease progression.

### 3.2. Responses and Resistance Associated With Anti-*BRAF* Therapy

Our patient received multiple lines of anti-*BRAF* therapy with response and resistance developing at different timepoints. Molecular analyses were performed to elucidate the mechanisms, and results were summarized in Tables 1 and 2. Perhaps due to the low tumor cellularity of the rebiopsy sample, no significant mutations were detected in the WES analysis.

*RNF43* mutations are detected in both primary and progression on doublet samples. *RNF43* mutations in MSS patients are loss-of-function (LOF) [15]. As *RNF43* degrades WNT pathway receptors, such LOF mutations cause accumulation of WNT receptors, resulting in over-signaling of WNT pathways which antagonizes MAPK-driven proliferation as demonstrated in in vitro studies [16]. Clinically, mutations in *RNF43* predicted improved response rates and survival outcomes in MSS patients, with median PFS in the MSS-*RNF43* mutated group being 10.1 months (versus 4.1 months in the MSS-*RNF43* wild-type group) [15]. These could explain our patient's initial durable response on doublet for 15 months, which superseded the study's PFS.

Although mutations in MAPK-related genes were widely reported as a resistance mechanism for anti-*BRAF* therapy [2, 17], these mutations were undetectable in our patient's samples. However, our WES results revealed mutations in PI3K and WNT pathways that cross-talk with the MAPK pathway, which could account for our patient's resistance mechanism while on doublet and triplet therapy [18, 19]. RICTOR, a component of mTORC2, is essential for the activation of the PI3K-AKT pathway and augmenting the oncogenic potential of receptor tyrosine kinases including EGFR [20]. The de novo *RICTOR* mutation during progression on doublet could cause activation of the PI3K-AKT pathway, leading to acquired resistance. NGS also revealed *PIK3CA E545K* as a subclonal true variant (0.36%) during progression on doublet, but it was not detected in tissue WES. Such discordance of mutations between liquid biopsy and tumor tissue has previously been reported and could be attributed to factors including low tumor fractions and intra-tumoral heterogeneity. Nevertheless, *RICTOR* and *PIK3CA* are both involved in the PI3K-AKT-mTOR pathway, which could contribute to the patient's progression on anti-*BRAF* therapy.

Despite disease progression on triplet, we rechallenged our patient with combinatory chemotherapy + bevacizumab, which halted disease progression in our patient transiently and he had static disease for 3 months. This is in line with the findings by Germani et al., where an additional benefit in PFS and postprogression survival was reported in MSS patients receiving chemotherapy + bevacizumab as a continuation of care beyond progression on doublet [21].

## 4. Conclusion

Our case report demonstrates the application of an in-house designed ddPCR assay to track p*BRAF*% as a surrogate marker of disease burden. Longitudinal surveillance using this biomarker could predict MRD, nonresponse, and disease progression, which is concordant with, or even precedes traditional radiological and serological markers. Fragmentomics analysis during progression on doublet and triplet also demonstrates its potential as a marker for tumor surveillance, which could be a promising research field with larger scale training sets in the future.

We also highlight the genomic and molecular features associated with response and resistance towards anti-*BRAF* therapies through serial and paired analyses of tissue, liquid biopsy, and in vitro studies. Although our patient did not harbor typical MAPK mutations accounting for anti-*BRAF* resistance, molecular studies revealed PI3K pathway–related mutations, which could potentially be targetable by PI3K inhibitors. Future research in precision oncology, including novel diagnostic and surveillance technologies coupled with comprehensive characterization of the molecular and fragmentomics landscape, could shed more light on the mechanism of resistance to anti-*BRAF* therapies and guide timely treatment decisions for patients.

## Figures and Tables

**Table 1:** WES analysis results of our patient's tissue samples, showing genes relevant to *BRAF* resistance. Column of “progression on triplet” was omitted due to no detection of significant mutations.

**WES**	**Variant frequent allele (VAF)**	
	**Primary site biopsy**	**Liver biopsy (progression on doublet)**
*BRAF V600E*	37.00%	39.29%
*RNF43 P154L*	33.33%	27.78%
*CTNNB1 T1161A N387K*	21.70%	15.84%

**Table 2:** Plasma NGS results of our patient's samples at progression on doublet and triplet, respectively.

**Plasma NGS**		**Progression on doublet**	**Progression on triplet**
Allele fraction (%)	*BRAF V600E*	3.44%	34.81%
	*CDH9 T737M*	0.31%	5.99%
	*PIK3CA E545K*	0.36%	0.19%
	*TP53 D281N D270N*	1.01%	20.21%

CNV amplifications (CNV score)	*EGFR*	No detection	Detected

## Data Availability

All relevant data are included within the article. Further inquiries can be directed to the corresponding author.
